# New application of anti-TLR monoclonal antibodies: detection, inhibition and protection

**DOI:** 10.1186/s41232-018-0068-7

**Published:** 2018-07-03

**Authors:** Ryutaro Fukui, Yusuke Murakami, Kensuke Miyake

**Affiliations:** 1 Division of Innate Immunity, Department of Microbiology and Immunology, The University of Tokyo, 4-6-1 Shirokanedai, Minato-ku, Tokyo 108-8639 Japan; 2 Department of Pharmacotherapy, Research Institute of Pharmaceutical Sciences, Musashino University, 1-1-20 Shin-machi, Nishitokyo-shi, Tokyo 202-8585 Japan; 3 Laboratory of Innate Immunity, Center for Experimental Medicine and Systems Biology, The Institute of Medical Science, The University of Tokyo, 4-6-1 Shirokanedai, Minato-ku, Tokyo 108-8639 Japan

**Keywords:** Toll-like receptor, Monoclonal antibody, Inhibitory antibodies, Inflammation, Autoimmune

## Abstract

Monoclonal antibody (mAb) is an essential tool for the analysis in various fields of biology. In the field of innate immunology, mAbs have been established and used for the study of Toll-like receptors (TLRs), a family of pathogen sensors that induces cytokine production and activate immune responses. TLRs play the role as a frontline of protection against pathogens, whereas excessive activation of TLRs has been implicated in a variety of infectious diseases and inflammatory diseases. For example, TLR7 and TLR9 sense not only pathogen-derived nucleic acids, but also self-derived nucleic acids in noninfectious inflammatory diseases such as systemic lupus erythematosus (SLE) or hepatitis. Consequently, it is important to clarify the molecular mechanisms of TLRs for therapeutic intervention in these diseases. For analysis of the molecular mechanisms of TLRs, mAbs to nucleic acid-sensing TLRs were developed recently. These mAbs revealed that TLR7 and TLR9 are localized also in the plasma membrane, while TLR7 and TLR9 were thought to be localized in endosomes and lysosomes. Among these mAbs, antagonistic mAbs to TLR7 or TLR9 are able to inhibit in vitro responses to synthetic ligands. Furthermore, antagonistic mAbs mitigate inflammatory disorders caused by TLR7 or TLR9 in mice. These results suggest that antagonistic mAbs to nucleic acid-sensing TLRs are a promising tool for therapeutic intervention in inflammatory disorders caused by excessive activation of nucleic acid-sensing TLRs. Here, we summarize the molecular mechanisms of TLRs and recent progresses in the trials targeting TLRs with mAbs to control inflammatory diseases.

## Background

Toll-like receptors (TLRs) recognize molecular patterns of pathogens and induce immune responses to protect host from pathogens. Although TLRs play important roles in the frontline of host defense, TLRs also recognize host-derived molecular patterns as a danger signal and lead to inflammation without infection. Given that the endogenous ligands of TLRs can be considered as metabolites, TLR responses to endogenous metabolites are likely to be under the homeostatic control [1]. Dysregulation of the interaction between TLRs and endogenous metabolites is thought to drive inflammation in a variety of diseases such as autoimmune diseases, metabolic syndrome, and heart failure. Consequently, it is important to ask whether TLRs are targets for therapeutic intervention in these diseases.

To investigate the role of TLRs in a variety of diseases, mAbs to TLRs are of great importance. For example, the mAb to mouse TLR3, 7, and 9 have shown that endogenous these TLRs are expressed on the cell surface as well as in intracellular compartments. Furthermore, several mAbs to TLRs are able to intervene TLR responses. An anti-TLR7 mAb is able to inhibit TLR7 responses in vitro and mitigate TLR7-dependent inflammation in vivo [2, 3]. In this review, we describe the molecular mechanisms by which TLRs drive disease progression and our trials to control TLR-dependent inflammatory disorders by anti-TLR mAbs.

### Overview of the molecular mechanisms of toll-like receptors

**Fig. 1:**
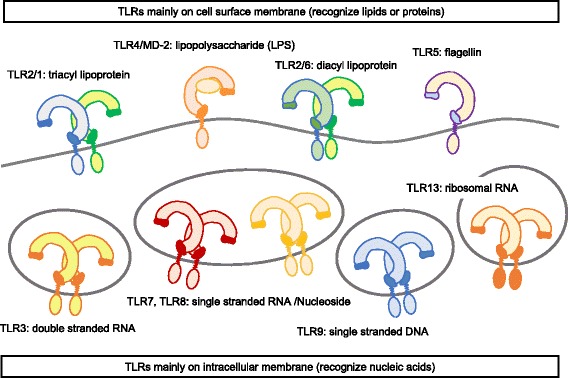
Subcellular distribution and ligands of TLRs. TLR2/TLR1 heterodimer, TLR2/TLR6 heterodimer, TLR4/MD-2 complex, and TLR5 are mainly localized on plasma membrane and recognize lipids or proteins. TLR3, TLR7, TLR8, and TLR9 are mainly localized on the membrane of intracellular particle and recognize nucleic acids

Toll-like receptors (TLRs) are a family of innate immune sensors, recognizing pathogen-associated molecular patterns (PAMPs) to activate immune response as a frontline of immune system [4–7]. TLR was found as a mammalian homolog of TOLL, the dorsoventral regulatory molecule of drosophila [8, 9]. The number of TLR varies with each species, for example, 10 TLRs are expressed in human whereas 12 TLRs are encoded in mice. Each TLR recognizes distinct PAMPs on the cell membrane of immune cells, and classified into two groups, depending on their ligands and cellular distribution. The cell surface TLRs, consisting of TLR1, TLR2, TLR4/MD-2, TLR5, and TLR6, recognize lipid or protein derived from bacteria (Fig. 1). The other group, consisting of TLR3, TLR7, TLR8, TLR9, and TLR13, respond to nucleic acids in endolysosome (Fig. 1).

All TLRs are categorized in type I transmembrane protein. TLRs recognize the ligands at the extracellular domain consisting of multiple leucine rich repeats to activate downstream signaling pathway via intracellular C-terminal region called Toll/interleukin-1 receptor (TIR) domain [10]. After the recognition of ligand, the conformation of TLR is changed and adapter proteins are recruited to the TIR domain [11–16]. Adapter proteins initiate the signal cascade to activate nuclear factor-kappa B (NF-κB) or interferon-regulatory factor (IRF), which promotes transcription of mRNA encoding cytokines/interferons [17–20].

### PAMPs and DAMPs, recognized by TLRs

Although the main role for TLRs is the recognition of PAMPs to protect host from pathogens, TLRs also recognize host-derived molecules [21, 22]. These molecules are called damage/danger-associated molecular patterns (DAMPs), released from damaged or dead cells. DAMPs are recognized by innate immune sensors and induce inflammation to facilitate the clearance of damaged cells by phagocytes [23]. If the damage is not restored, DAMPs are continuously released and recognized by innate immune sensors. The cells expressing innate immune sensors strongly produce inflammatory cytokines, and the cytokines increase tissue damages, which in turn promote release of more DAMPs, and finally, a vicious circle is constructed among DAMPs, innate immune sensors and inflammation.

DAMPs are recognized by innate immune sensors because of the similarity of the structure to PAMPs. Recently, many types of DAMPs are found as endogenous ligands of TLRs [24, 25]. For example, TLR4/MD-2 recognizes bacteria-derived lipopolysaccharide (LPS) and host-derived fatty acids as PAMPs and DAMPs, respectively [26–28]. It is well known that fatty acid is a mediator of metabolic syndrome, and obesity is a condition where the response of TLR4/MD-2 is out of control. In obese adipose tissues, production of chemokines, such as monocyte chemoattractant protein-1 (MCP-1) is enhanced, and macrophages with C-C chemokine receptor type 2 (CCR2), the receptor of MCP-1, infiltrate into adipose tissues [29–31]. Infiltrated macrophages closely interact to adipocytes and strongly recognize saturated fatty acids released from adipocytes. As result, large amounts of cytokines are released from macrophages and vicious circle of inflammation is constructed by obesity.

DAMPs induce inflammation and diseases, but given that DAMPs are generated and released by metabolism, inflammation induced by DAMPs is a homeostatic responses. Excessive activation of innate immune sensors by DAMPs induces chronic inflammation and irreversible tissue damage. This concept is proposed as “homeostatic inflammation” [1, 32].

### Controlling system of toll-like receptors

**Fig. 2:**
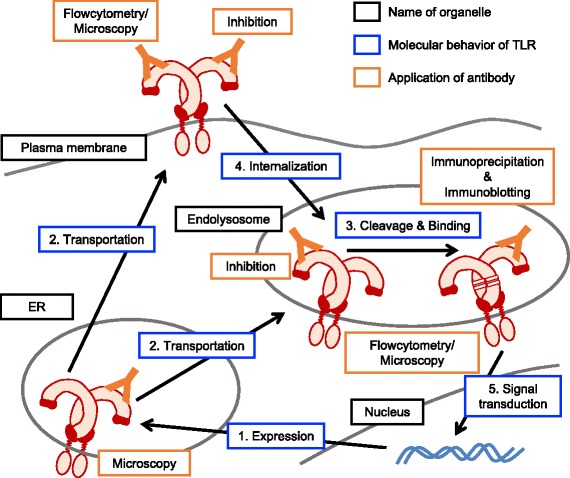
Application of anti-TLR for the analysis of molecular mechanisms of TLR. Expressed TLRs are transported from ER to plasma membrane or endolysosome (1 and 2). In endolysosome, nucleic acid-sensing TLRs are cleaved and the N-terminal fragment binds to the C-terminal fragment (3). After the modification, TLR recognize ligand and signaling pathway is activated (5). TLRs on cell surface recognize ligand and activates signaling pathway. Some of them are internalized by the recognition of ligand or spontaneous trafficking (4). For the analysis of these molecular behaviors of TLR, monoclonal anti-TLR is well exploited. Amount and expression pattern among cell types are analyzed by flowcytometry. Subcellular distribution is observed by immunofluorescence microscopy. Alternatively, flowcytometry following the immunostaining with or without detergent is able to discriminate the distribution between cell surface and intracellular particle. Proteolytic cleavage and binding of nucleic acid-sensing TLR are detected by immunoprecipitation and immunoblotting. In addition to these application for detection, several antibodies have inhibitory effect on the response of TLRs. Words in black frames, blue frames, and orange frames are the name of organelle, the molecular behavior of TLR, and the application of anti-TLR, respectively

To avoid excessive homeostatic inflammation, there are multiple controlling systems for TLRs varying from gene transcription to protein degradation (Fig. 2). Especially, the responses of nucleic acid-sensing TLRs should be strictly controlled because the structure of nucleic acid is highly conserved between pathogens and hosts. TLR7, recognizing single-stranded RNA and guanosine analogs, is one of the well-known pathogenic factors of autoimmune disease model. For example, systemic lupus erythematosus (SLE)-like phenotype is spontaneously induced in BXSB^*Yaa*^ mice, which harbor an additional copy of the *Tlr7* gene on Y chromosome (Table 1) [33–35]. *Tlr7* is originally encoded on the X chromosome, so that BXSB^*Yaa*^ mice express 2 copies of TLR7 and the response to endogenous TLR7 ligand is enhanced.

**Table 1:** Relation of TLRs with chronic inflammatory diseases

Disease	TLR	Roles for TLR on disease
Nonalcoholic steatohepatitis (NASH)	TLR4 TLR9	*TLR4* mRNA expression is increased in the liver of NASH patients [49]. TLR9 recognizes host-derived mitochondrial DNA and induces inflammation [47, 48].
Psoriasis	TLR7 TLR9	Antimicrobial peptide LL37, a factor of psoriasis, associates with self-derived DNA and stimulate TLR9 in pDCs [67]. Administration of imiquimod, a TLR7 ligand, induces psoriasis-like dermatitis via T cell activation with IL-17/IL-23 production [71] .
Systemic lupus erythematosus (SLE)	TLR7 TLR9	TLR7 and TLR9 contribute to the production of autoantibody in SLE model mouse [34, 35, 72]. Especially, TLR7 is thought as an inducer of the phenotype [34, 35, 73, 74]. Controversially, protective effect of TLR9 on SLE is also reported [73, 75, 76].
Celiac Disease	TLR7 TLR8	Suggestive association of the SNP on *TLR7* and *TLR8* with celiac disease is found by genome-wide association study (GWAS) [77].
Type 1 diabetes (T1D)	TLR4 TLR9	TLR4 expresses on β cells in islets and induces signaling by recognizing HMGB1 [78]. TLR9 contributes to the activation of T cells in NOD mice, a model of T1D [69, 79, 80].
Rheumatoid arthritis	TLR8 TLR9	Human TLR8 transgenic mice develop arthritis spontaneously [81]. Cathepsin K is required for the response of TLR9, and an inhibitor of Cathepsin K attenuates T_H_17 polarization and arthritis [68].

Not only the amount of TLR7, the controlling molecules of TLR7 is also important to avoid lethal inflammation [36]. Unc93 homolog B1 (UNC93B1) is one of the essential molecules for the function of nucleic acid-sensing TLRs. Furthermore, UNC93B1 balances the responses of nucleic acid-sensing TLRs [37]. UNC93B1 binds to TLRs in the ER and transport them to endolysosome where TLRs are matured by the proteolytic cleavage of the extracellular domain [38–40]. These function of UNC93B1 is abolished by the deletion or mutation of C-terminal region, while the deletion or mutation of N-terminal region collapse the balance of the responses between TLR7 and TLR9 [37, 41, 42]. In detail, the region around 34th aspartic acid (D34) is responsible for the regulation of the responses of TLR7 and TLR9, so that the alanine mutation of the D34 (D34A mutation) enhances the response of TLR7 and declines the response of TLR9. The mice harboring D34A mutation on *Unc93b1* (D34A mice) suffer from systemic inflammation, such as hepatitis, thrombocytopenia, splenomegaly and nephritis, depending on the excessive response of TLR7 [43, 44].

These TLR7-dependent disease models might develop inflammation by recognizing endogenous TLR7 ligand, such as single-stranded RNA and guanosine analogs [45, 46]. Like TLR7, TLR9 recognizes endogenous DNA ligand and also contributes to the inflammatory disease (Table 1). TLR9 recognizes mitochondrial DNA (mtDNA) released from hepatocyte and drives nonalcoholic steatohepatitis (NASH) [47, 48]. NASH is a common liver diseases characterized by fatty liver, in which TLR9 plays a pathogenic role in driving inflammatory response by responding to mtDNA released from damaged hepatocytes [49].

### Anti-TLR mAbs for the analysis of molecular mechanisms in TLRs

Given that TLRs contribute to the homeostatic inflammation and diseases, analysis of the molecular mechanisms of TLR is very important to develop therapeutic strategy. Monoclonal anti-TLRs have been established to detect endogenous TLRs from the early years of TLR research and many of new findings were brought. We also exploited mAbs for the studies of TLRs by establishing anti-murine TLR1, TLR2, TLR3, TLR4/MD-2, TLR5, TLR6, TLR7 and TLR9 **(**Table 2 and Fig. 2**)** [3, 50–54]. For example, anti-TLR4/MD-2 complex monoclonal antibody reveals the direct binding of LPS to TLR4/MD-2, which is confirmed by the analysis of crystal structure later [55–57].

**Table 2:** Application of anti-monoclonal antibodies we established

TLR (mouse)	Name of clone	Application	Reference
TLR1	TR23	FC, IP	[50]
TLR2	CB225	FC, IP	[50]
TLR4/MD-2	MTS510 Sa15–21	FC, IP, BL (in vitro) FC, IP, BL (in vivo)	[54, 57, 64]
TLR6	C1N2	FC, IP	[50]
TLR5	ACT5	FC, IP	[51, 82]
TLR3	CaT3 PaT3	FC, IP FC, IP	[52]
TLR7	A94B10	FC, IP, IF, BL	[2, 3, 43]
TLR9	J15A7 B33A4 C34A1 NaR9	FC, IP, IF FC, IP FC, IP, IF FC, IP, IF, BL	[53, 66]

*FC* flowcytometry, *IP* immunoprecipitation, *IF* immunofluorescence microscopy, *BL* blocking

Anti-TLR mAbs also reveal the subcellular distribution of TLRs. As shown in Fig. 1, it was believed that TLR1, TLR2, TLR4/MD-2, and TLR6 are localized on cell surface, and TLR3, TLR7, and TLR9 are localized on intracellular vesicles. It is true and important for TLRs to be functional, but endogenous intracellular TLR1, TLR2, TLR4/−MD-2 and TLR6 were detected by flowcytometry analysis with mAbs [50, 58, 59], and intracellular nucleic acid-sensing TLRs were detected on plasma membrane [2, 52, 53, 60].

Another important application of mAb is immunoprecipitation for immunoblotting. Concentration by immunoprecipitation is required for detection of endogenous TLR because the amount of endogenous TLR is not enough to detect by the immunoblotting of whole cell lysate. Even if TLRs are overexpressed, non-tagged TLR or the fragments of TLR generated by proteolytic cleavage is detectable by anti-TLR mAbs. We found that TLR9 is cleaved in endolysosome and separated to N-terminal and C-terminal fragments by using anti-TLR9 mAb [53]. Previous reports suggested that the cleaved C-terminal region of TLR9 is functional with the cleaved N-terminal region serving as a negative regulator [61, 62]. We established two types of anti-TLR9 mAbs to detect the both of fragments of TLR9; one clone is bound to N-terminal region and the other is bound to C-terminal region [53]. By using these mAbs, we clarified that the N-terminal region of TLR9 binds to the C-terminal region, and the binding is essential for the response of TLR9. Not only TLR9, the requirement of proteolytic cleavage and the binding of the fragments are also confirmed in TLR3 and TLR7 with monoclonal antibodies [3, 52, 63].

### Inhibitory effect of anti-TLR mAbs

Among anti-TLR mAbs, several clones inhibit the response of cells to TLR ligand. For examples, mAbs to human TLR3 (clone TLR3.7), mouse TLR4/MD-2 (clone MTS510), mouse TLR7 (clone A94B10), and mouse TLR9 (clone NaR9) inhibit the response of target TLR [2, 43, 64, 65]. Interestingly, the responses of intracellular TLRs, such as TLR3, TLR7 and TLR9, are inhibited by the monoclonal antibody in culture medium. Although these TLRs are also localized on cell surface as described above, they recognize and signal nucleic acids in endolysosome. How do these inhibitory anti-TLR mAbs reach to intracellular TLRs?

In case of TLR7, a monoclonal anti-TLR7 “A94B10” binds to cell surface TLR7 and accumulated in endolysosomes as immune complex [2]. A part of the antibody uptake depends on Fc receptor, but the uptake of A94B10 remains without Fc receptor. Furthermore, the uptake of A94B10 by the cells from *Tlr7*^−/−^ mice or UNC93B1 deficient mice is attenuated. Since UNC93B1 is essential for the cell surface localization of TLR7, inhibitory function of A94B10 might depend on cell surface TLR7.

A monoclonal anti-TLR9, clone “NaR9” also inhibits TLR9 responses. A94B10 inhibits the response of TLR7 in various types of cells, such as bone marrow-derived macrophages (BM-MCs), BM-conventional dendritic cells (BM-cDCs), BM-plasmacytoid DCs (BM-pDCs), and B cells, whereas NaR9 only inhibits the response of TLR9 in BM-MCs and BM-cDCs [66]. As a mechanism behind the difference between these two mAbs remains unclarified, NaR9 is internalized by BM-MCs and BM-cDCs but not by pDCs. Interaction between mAb and cell surface TLR7 or TLR9 and subsequent internalization are likely to be required for the inhibitory effect of these mAbs.

### New application of anti-TLR mAbs for therapeutic intervention

If an inhibitory anti-TLR mAb is functional in vivo, the antibody is able to be applied to the treatment of inflammatory diseases induced by TLRs (Table 1). Among the antibodies mentioned above, an anti-TLR9 NaR9 rescues the mice from TLR9-dependent lethal experimental hepatitis [66]. Although the effect of NaR9 on the chronic inflammatory disease is unknown, pre-treatment of NaR9 significantly reduce the inflammatory cytokine production and acute hepatitis induced by D-(+)-galactosamine and TLR9 ligand CpG-ODN. These results suggest that NaR9 is able to inhibit TLR9-dependent hepatitis. As TLR9 is suggested to drive inflammation in NASH, anti-TLR9 mAb may be promising for therapeutic intervention in NASH or other diseases such as psoriasis, rheumatoid arthritis, and type 1 diabetes (Table 1) [47, 67–69].

Anti-TLR4/MD-2 monoclonal antibody, Sa15–21 also rescues the mice from lethal experimental hepatitis induced by D-(+)-galactosamine and TLR4 ligand LPS [64]. Interestingly, Sa15–21 does not directly inhibit the response of TLR4/MD-2, on contrary, enhances the response of TLR4/MD-2 and increase the production of TNF-α. In fact, Sa15–21 induces antiapoptotic genes via TLR4/MD-2 with agonistic effect and protects mice from lethal hepatitis.

For the treatment of inflammatory disease, an inhibitory anti-TLR7 monoclonal antibody, clone A94B10 is effective in the systemic inflammation caused by TLR7 hyper-response [2]. As mentioned above, the mice harboring the D34A mutation on UNC93B1 (D34A mice) suffer from severe inflammation with TLR7-dependent manner. The administration of A94B10 to D34A mice significantly attenuates the phenotypes, such as splenomegaly, thrombocytopenia, autoantibody production, and hepatitis with fibrosis of liver. It is important to distinguish whether the effect of antibody is therapeutic or prophylactic. Even if the administration of A94B10 was started when D34A mice had already developed thrombocytopenia, the anti-TLR7 mAb was still effective. These results suggest that anti-TLR7 is a promising candidate of therapeutic drug to cure TLR7-dependent autoimmune diseases, for examples, SLE or autoimmune hepatitis.

## Conclusion

Homeostatic inflammation induced by TLRs is tightly related to health and disease. To investigate the molecular mechanisms of TLRs, monoclonal antibody is an essential tool for experiment. In these 2 decades, many researchers have revealed a variety of the molecular properties of TLRs with anti-TLR monoclonal antibodies. Moreover, the application of anti-TLR monoclonal antibody is expanding to therapeutic intervention. There is no evidence of the therapeutic effect of anti-TLR on the disease in human, however, TLR antagonist nucleic acids has already proceeded to clinical trial [70]. If the trial of nucleic acid medicine is successful, development of anti-human TLR mAbs will also be a promising way to control TLR-dependent diseases. Of course, the application of anti-TLR mAbs might compete nucleic acid medicine, but mAbs have advantages of higher specificity, lower off-target effect, and longer half-life comparing to nucleic acids. Not only for clinical application, anti-human TLR should be continuously established for the analysis of the molecular mechanism of human TLRs.

## Abbreviations

BM-cDC: bone marrow-derived conventional dendritic cell
BM-MC: bone marrow-derived macrophage
BM-pDC: bone marrow-derived plasmacytoid dendritic cell
CCR2: C-C chemokine receptor type 2
D34A mutation: alanine mutant of 34th aspartic acid in UNC93B1
DAMPs: damage/danger-associated molecular patterns
IRF: interferon-regulatory factor
mAb: monoclonal antibody
MCP-1: monocyte chemoattractant protein-1
mtDNA: mitochondrial DNA
NASH: nonalcoholic steatohepatitis
NF-κB: nuclear factor-kappa B
PAMPs: pathogen-associated molecular patterns
SLE: systemic lupus erythematosus
TIR domain: Toll/IL-1 receptor domain
TLR: Toll-like receptor
UNC93B1: Unc93 homolog B1
